# Epidemiologic Trends, Genetic Diversity, and Risk Factors of Norovirus Outbreaks in Beijing, China, 2016−2022

**DOI:** 10.3390/v18030295

**Published:** 2026-02-28

**Authors:** Yu Wang, Weihong Li, Baiwei Liu, Lingyu Shen, Yi Tian, Lei Jia, Hanqiu Yan, Jing Zeng, Qingbin Lu, Daitao Zhang, Peng Yang, Quanyi Wang, Zhiyong Gao, Fuqiang Cui

**Affiliations:** 1 Institute for Infectious Disease and Endemic Disease Control, Beijing Center for Disease Prevention and Control, Beijing 100013, China; wangyuyu@bjmu.edu.cn (Y.W.);; 2 Beijing Key Laboratory of Surveillance, Early Warning and Pathogen Research on Emerging Infectious Diseases, Beijing Center for Disease Prevention and Control, Beijing 100013, China; 3 Department of Laboratory Science and Technology and Vaccine Research Center, School of Public Health, Peking University, Beijing 100191, China; 4 Beijing Research Center for Respiratory Infectious Diseases, Beijing 100013, China; 5 Department of Epidemiology and Biostatistics, School of Public Health, Peking University, Beijing 100191, China; 6 Key Laboratory of Epidemiology of Major Diseases, Peking University, Ministry of Education, Beijing 100191, China

**Keywords:** norovirus, disease outbreaks, public health, prevalence trends, genotype

## Abstract

Norovirus is the leading cause of acute gastroenteritis outbreaks worldwide. A total of 1859 norovirus outbreaks were reported from 2016 to 2022 in Beijing, China. GII.2[P16] was the predominant genotype during 2016–2021, and GII.3[P12] during 2021–2022. In the early stage of the COVID-19 epidemic (January to June of 2020), strict prevention and control measures were implemented, and only eight norovirus outbreaks were reported. Most norovirus outbreaks occurred in schools (95.6%, 1778). As the level of schooling rises, the scale of norovirus outbreaks also increases (median case numbers: 8 for kindergarten, 10 for primary school, 11 for secondary school, and 14 for college; *p* trend < 0.001), while the attack rate decreases (median attack rates were correspondingly 25.8%, 17.5%, 10.0%, and 8.8%; *p* trend < 0.001). Compared to outbreaks caused by person-to-person transmission, foodborne and waterborne outbreaks are larger in scale. Delayed outbreak response is also a risk factor for larger-scale outbreaks. Norovirus outbreaks have emerged as a significant concern for public health in Beijing. Persistent genotyping efforts are essential to facilitate early warning. Outbreaks in different locations or through different transmission routes require specific prevention and control measures.

## 1. Introduction

Noroviruses are the main pathogen leading to a great disease burden of acute gastroenteritis (AGE) outbreaks and sporadic cases [1,2,3]. The symptoms of norovirus infection mainly manifest as acute gastroenteritis, characterized by vomiting or diarrhea, but it can also cause a small number of people to develop severe illness and even death [4,5]. Current studies have shown that norovirus infections usually occur in cold seasons, which is quite different from bacterial enteric infections [6]. In addition, norovirus could spread easily in semi-closed environments and often lead to clusters and outbreaks [7,8]. This triggers panic in the public, disrupts the normal order in families or public places, and poses certain challenges to social governance in mega-cities like Beijing.

Previous studies have shown that the majority of AGE outbreaks in Beijing were caused by norovirus [9], and these norovirus outbreaks exhibited similar characteristics to those reported in the whole of China [2], but exhibited different characteristics, such as outbreak-associated settings and the dominant genotypes, compared to those reported in other countries [10,11,12,13]. Norovirus outbreaks were mainly reported in schools, and the GII.2[P16] strain predominated in recent years in Beijing, while long-term care facilities (LTCF) were the most reported outbreak settings, and GII.4 was the dominant genotype abroad [10,11,12,13]. Although the prevalent genogroup in norovirus outbreaks was predominantly GII, there were variations in the circulating genotypes in Beijing. The predominant genotype in norovirus outbreaks was GII.4 before 2014, followed by GII.17[P17] in 2014–2015, and more recently GII.2[P16] in 2016–2017 [9], which was consistent with the genotypic trend of norovirus outbreaks reported in China as a whole [2,14]. More recently, between 2023 and 2025, GII.17[P17] has resurged as a causative genotype of norovirus outbreaks—not only in Beijing or other provinces in China [15,16], but also in other regions worldwide [17,18]. This observation underscores the genetic variation characteristics of noroviruses and the critical importance of continuous genotype analysis. Furthermore, current studies have indicated that norovirus outbreaks with different genotypes presented various epidemiological characteristics. A study in Spain found that GII.4 Sydney 2012 outbreaks were significantly associated with nursing homes, while GII.2[P16] and GI.3[P3] were most frequently identified in youth hostels/campgrounds [10]. Our previous study showed that foodborne transmission was more common in GII.17[P17] outbreaks than in GII.2[P16] ones [9]. However, these studies were based on insufficient sample sizes of norovirus outbreaks and had a relatively short time span; thus, further evidence is required to elucidate the genotype-specific outbreak characteristics. Studies also have shown that the genotype was associated with the occurrence and attack rate of norovirus outbreaks. The GII.13[P16] outbreaks had a higher total attack rate [19], and infection with genotype GII.4 tended to be associated with norovirus outbreaks in hospital settings [20]. Other factors, including vomiting, shared activities in public areas, outbreak timing, location, setting, and transmission route, were also related to the scale or attack rate of norovirus outbreaks [19,21,22]. However, research in this area remains limited, and more comprehensive analyses are needed.

Recently, a few studies have investigated the epidemiological characteristics of norovirus outbreaks with different genotypes, with most relevant research focusing on single-genotype analyses [15,23,24]. Although preliminary research on the general characteristics of norovirus outbreaks has been conducted in Beijing [9], these investigations are relatively dated. Furthermore, Beijing has a highly sensitive norovirus surveillance system: the local outbreak definition (≥3 epidemiologically linked cases occurring within 3 days) is implemented with enhanced surveillance capacity that enables more timely and comprehensive detection of outbreaks, thus yielding high-quality surveillance data. Given this, an in-depth analysis of the incidence trends, epidemiological features, and genotype-specific characteristics of norovirus outbreaks in Beijing will not only address the aforementioned research gaps but also provide a valuable reference for the implementation of norovirus prevention and control work in other regions.

## 2. Materials and Methods

### 2.1. Data Source 

Data was obtained from Beijing Center for Disease Control and Prevention (Beijing CDC). Beijing CDC was responsible for the surveillance of norovirus and the investigation of norovirus outbreaks in Beijing municipality. Detailed information about the surveillance and the data collection procedures was described in our previous studies [9]. The case detection, case definition, reporting channels or reporting threshold, and investigation protocols were uniform throughout the study period. The laboratory diagnostic methods and testing reagents used remained standardized. No modifications were made to the surveillance scope or data collection protocols that would have affected surveillance sensitivity. 

### 2.2. Inclusion and Exclusion Criteria of Outbreaks

A norovirus outbreak was defined as an outbreak with three or more epidemiologically linked AGE cases within three days, and at least two of these cases tested positive for norovirus on laboratory examination. AGE was defined as the development of diarrhea (three or more loose stools) or vomiting (one or more episodes) within 24 h. The norovirus outbreaks reported between 1 September 2016 and 31 August 2022 were included. 

### 2.3. Data Collection

The information collected on norovirus outbreaks included onset of outbreak, occurrence area, type of outbreak setting, case number, attack rate, transmission mode, norovirus genogroup and genotype, and the date when the response was initiated by health departments. 

We defined a year from September 1 to August 31 of the following year, according to the epidemic characteristics of norovirus outbreak in Beijing. The meteorological season was divided into four categories: spring (March–May), summer (June–August), autumn (September–November), and winter (December–February). Three occurrence areas were classified into urban, semi-urban, and rural [9]. Outbreak setting was divided into five categories: kindergartens, primary schools, secondary schools, colleges, and other settings (such as companies or institutions, residential communities, hospitals, nursing homes, hotels). 

Transmission mode was categorized as person-to-person, foodborne, waterborne and unknown. The transmission mode was mainly determined by field experts through comprehensive analysis of detailed epidemiological investigations of individual cases, hygienic investigations on the outbreak settings, epidemiological curves of outbreaks, and laboratory test results. In cases where an outbreak involved multiple transmission modes, the one that caused and leaded to norovirus transmission was designated as the transmission mode of the outbreak. 

The time period of coronavirus disease 2019 (COVID-19) was divided into three phases: “before COVID-19” (1 September 2016 to 31 December 2019); “extremely strict policy against COVID-19” (1 January 2020 to 30 June 2020), during which extremely strict measures were taken, such as city lockdown, schools were closed and implemented online teaching, which resulted in a widespread decline in social activity; and “strict policy against COVID-19” (1 July 2020 to 31 August 2022), during this stage, social activity gradually began to resume, the measures adopted were more targeted and less extensive, with the minimum impact on the lives of the public. 

Timeliness of outbreak response was the time interval between onset date of the first case and the outbreak reporting date, which was classified as early (within 1 day), medium (2–3 days), and late (more than 3days). The scale of norovirus outbreaks was divided into two levels: large scale outbreak was defined as one involving 15 or more cases; small scale outbreaks was defined as those with less than 15 cases. The attack rate of norovirus outbreaks was divided into two levels: high attack rate was defined as 30% or greater, and low attack rate was defined as less than 30%. The classification criterion was based on the distribution characteristics of both the scale and attack rate of norovirus outbreaks reported in Beijing, with the 75th percentile (P75) of each indicator adopted as the threshold. For an outbreak, the denominator of the attack rate refers to the number of people who are at risk of norovirus infection—for example, the number of people who consumed a specific meal in a foodborne outbreak, or those who had contact with cases, their vomit, or their diarrhea in a person-to-person transmission outbreak. 

### 2.4. Statistical Analysis

Categorical variables were presented as counts and percentages (%), while continuous variables were presented as median and interquartile range (IQR). Differences in the distribution of categorical variables between groups were compared using the chi-square test or Fisher’s exact test. Univariable analysis was conducted initially, with variables significant at *p* < 0.1 retained as candidate variables. Final variable selection for the multivariable model was based on a comprehensive integration of univariable results and epidemiological relevance. Backward elimination was performed to establish a final model retaining those with *p* <0.05 in the model. Prior to multivariable logistic regression analysis, potential multicollinearity among candidate variables was evaluated using variance inflation factors (VIF) and GVIF. Additionally, potential epidemiologically meaningful interactions between key variables were tested in the multivariable model to explore their combined effects on the outcome. Data sorting and analysis were performed using R software (version 4.2.1, R Foundation for Statistical Computing, Vienna, Austria).

## 3. Results

### 3.1. Overview of Norovirus Outbreaks from 2016 to 2022

From 2016 to 2022, 3333 AGE outbreaks were reported in Beijing, among which 1859 (55.8%) were caused by norovirus. And 24,736 cases were identified in these norovirus outbreaks. The median outbreak size was nine cases (interquartile range, IQR 6–15, range 3–389), and the median attack rate was 21.4% (IQR 12.5–31.7%, range 0.3–100.0%).

**Figure 1:**
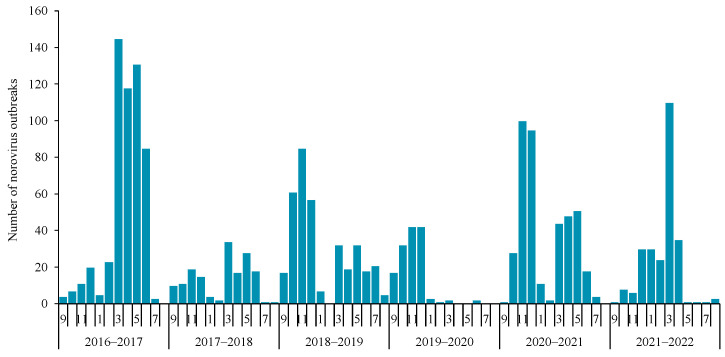
Temporal distribution of norovirus outbreaks reported in Beijing, China, 2016–2022.

Temporal distribution characteristics. The number of reported norovirus outbreaks varied over the years, with the highest number noted in 2016–2017 (29.7%, 552). In 2016–2017, 2017–2018, and 2018–2019, the monthly average reported number of norovirus outbreaks was 46, 13, and 30, respectively. In the very early stage of the COVID-19 epidemic (January to June of 2020), only eight norovirus outbreaks were reported. However, in 2020–2021 and 2021–2022, the monthly average reported number of norovirus outbreaks was 34 and 21, respectively. The monthly reported number of norovirus outbreaks is shown in Figure 1.

The norovirus outbreaks mainly occurred in spring (45.6%, 847), while only 181 outbreaks were reported in summer. The median case number and attack rate of norovirus outbreaks in 2020–2021 were lower than those of the other five years. The number, scale, and attack rate of reported norovirus outbreaks in each surveillance year and season are shown in Table 1.

Spatial distribution characteristics. The majority of norovirus outbreaks (58.1%, 1080) were reported in urban districts. Most norovirus outbreaks occurred in schools (95.6%, 1778), especially in kindergarten (53.9%, 958/1778) and primary school (37.3%, 664/1778). There were only two norovirus outbreaks reported in nursing homes and eight in hospitals. As the level of schooling rises, the scale of norovirus outbreaks increases (*p* trend < 0.001), while the attack rate decreases (*p* trend < 0.001). The number, scale, and attack rate of reported norovirus outbreaks in each occurrence area and outbreak setting are shown in Table 1.

Transmission mode. Among 1811 norovirus outbreaks with known transmission mode, the majority were person-to-person outbreaks (96.7%), 54 were foodborne, and only 6 were waterborne.

### 3.2. Molecular Epidemiological Characteristics of Norovirus Outbreaks

Norovirus genogroups. Among 1857 norovirus outbreaks with known genogroup information, 85.6% (1589) were GII, 9.6% (179) were GI, and 3.0% (56) were GI/GI mixed; the other 33 outbreaks were mixed infections by norovirus and other pathogens (mainly enteric adenovirus, sapovirus, and rotavirus). The distribution characteristics and variability of norovirus outbreaks by genogroup are presented in Table 2.

For the distribution of the year, 33.9% (538) of GII outbreaks were reported in 2016–2017, while the majority of GI outbreaks (32.4%) and GI/GII mixed outbreaks (53.6%) were reported in 2018–2019; *p* < 0.001. For the characteristic of season, the majority of GII outbreaks (44.9%) and GI outbreaks (57.0%) were reported in spring, while GI/GII mixed outbreaks were scattered across various seasons; *p* < 0.001. For the distribution of outbreak setting, GII outbreaks mainly occurred in kindergartens (55.3%), while the majority of GI outbreaks (57.0%) occurred in primary schools, and more GI/GII mixed outbreaks (32.1%) occurred in other closed settings; *p* < 0.001. For transmission mode, more GI/GII mixed outbreaks were transmitted through food and water (13.0%), compared with GI outbreaks (6.9%) and GII ones (2.4%); *p* < 0.001. There was no statistical difference in the regional distribution among norovirus outbreaks of these three kinds of genogroups.

**Figure 2:**
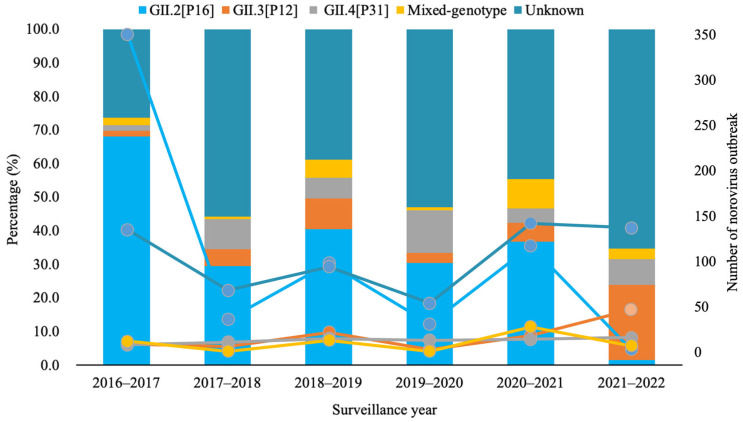
The major genotype distribution of norovirus outbreaks during 2016–2022 in Beijing, China.

Norovirus genotypes. Of the 1157 successfully dual-typed norovirus outbreaks, 54.9% (635) were GII.2[P16], followed by GII.3[P12] (9.1%, 105), GII.4[P31] (6.6%, 77), mixed-genotype (5.4%, 62), and others 24.0% (278). The distribution characteristic and variability of norovirus outbreaks by genotype are presented in Figure 2 and Table 2. For yearly distribution, GII.2[P16] outbreaks were mainly reported in 2016–2017 (55.1%), while GII.4[P31] outbreaks were almost evenly distributed over the six years (*p* < 0.001). For seasonal distribution, the majority of GII.2[P16] outbreaks (49.4%) and mixed-genotype ones (40.3%) were reported in spring, while GII.3[P12] outbreaks were mainly reported in winter (41.0%), and GII.4[P31] ones were mainly reported in autumn (45.5%) (*p* < 0.001). Though all four kinds of genotype outbreaks mainly occurred in kindergartens and primary schools, a higher proportion of mixed-genotype outbreaks (14.5%) occurred in other closed settings; *p* < 0.001. The main transmission mode was person-to-person, while a higher proportion of mixed-genotype outbreaks (6.6%) were foodborne (*p* = 0.01).

### 3.3. Risk Factors for the Scale of Norovirus Outbreaks

Factors of year, season, occurrence area, outbreak setting, transmission mode, genogroup, genotype, time periods of COVID-19, and timeliness of outbreak response were analyzed. Results are shown in Table 3. All factors presented in Table 3, except season and time period of COVID-19, were included in the multivariable logistic regression model. The number of norovirus outbreaks with a scale of more than 15 cases was 467, resulting in an event-per-variable (EPV) ratio of 67. Four factors remained significantly associated with the scale of the norovirus outbreak in the final model.

Year: In 2016–2017, 37.1% of reported norovirus outbreaks had a size exceeding 15 cases, which was higher than the proportion of outbreaks with more than 15 cases in 2019–2022. Outbreak setting: Compared with kindergartens, other kinds of settings had a higher risk of having norovirus outbreaks with more than 15 cases. Transmission mode: 74.1% of foodborne norovirus outbreaks and 83.8% of waterborne ones involved more than 15 cases each, with both proportions higher than that of norovirus outbreaks transmitted via person-to-person. Timeliness of outbreak response: For norovirus outbreaks with a medium response time (2–3 days), the proportion of those involving more than 15 cases reached 28.1%—higher than that for the early response time (≤1 day) group.

### 3.4. Risk Factors for the Attack Rate of Norovirus Outbreaks

Factors associated with the attack rate of norovirus outbreaks were the same as those in the analysis of outbreak scale, and the results are shown in Table 4. All factors presented in Table 4, except the season and the time period of COVID-19, were included in the multivariable logistic regression model. The number of norovirus outbreaks with an attack rate higher than 30% was 525, resulting in an EPV ratio of 75. Five factors remained associated with the attack rate of the norovirus outbreak in the final model.

Year: In 2016–2017, 35.0% of the reported norovirus outbreaks had an attack rate exceeding 30.0%—a higher proportion than that for 2020–2021 (21.4%) and 2021–2022 (26.7%). Occurrence area: 30.1% of norovirus outbreaks reported in urban districts had an attack rate exceeding 30.0%—higher than the 25.7% of those reported in semi-urban districts. Outbreak setting: Among norovirus outbreaks occurring in kindergartens, 39.2% had an attack rate exceeding 30.0%—a higher proportion than that for primary schools (18.1%), secondary schools (4.8%), and colleges (13.8%). Genogroup: For GII outbreaks, the proportion with an attack rate exceeding 30.0% reached 30.4%—higher than that for the GI/GII mixed ones (10.9%). Genotype: For GII.2[P16] outbreaks, the proportion with an attack rate exceeding 30.0% reached 33.6%—higher than that for GII.4[P31] outbreaks (26.0%).

## 4. Discussion

In this study, norovirus outbreaks reported over six consecutive years in Beijing were analyzed, and several new findings were identified. A biennial pattern was observed, with higher norovirus outbreak activity in 2016–2017, 2018–2019, and 2020–2021, and lower activity in the years between.A new strain of GII.2[P16] emerged in October 2016 in Beijing, and it soon led to a huge epidemic wave between February and June of 2017, demonstrating the high transmissibility of the new variant strain. This was also supported by evidence that a new variant of GII.2 led to excess norovirus-associated acute diarrhea cases by 152.6% [25]. Even though GII.2[P16] was still a major genotype in norovirus outbreaks during 2017 and 2021, the wave caused by this specific genotype was getting lower, compared with that during the first year of its predominance. The underlying mechanism might be the universal susceptibility to a new variant strain during its first appearance and subsequent development of immune memory against this variant, which restricts its transmission. The phenomenon of biennial pattern of high activity in norovirus outbreaks has been rarely reported, yet such a pattern has been observed in the seasonal patterns of norovirus infections in Denmark and Spain [10,26], as well as in hand-foot-and-mouth disease (HFMD) epidemics [27]. Whether the biennial pattern truly exists and the underlying driving factors require longer-term surveillance data and more in-depth analyses for verification.

GII.2[P16] was still the predominant strain, though it presented a waning trend in leading to epidemic waves. This was quite different from other countries, where GII.4 still prevailed in norovirus infections or outbreaks [10,11,12,13,28,29]. GII.2[P16] outbreaks once prevailed in Japan and Germany in 2016–2017 [30,31], but GII.4 returned to being the leading strain again in 2018 [32,33]. The difference in genotype prevalence might be related to the different focus of outbreak surveillance or differences in population susceptibility. However, it was worth noting that genotype distribution in norovirus outbreaks was different from that in sporadic cases of pediatric acute gastroenteritis in Beijing. GII.4 Sydney [P31] predominated among outpatient children with diarrhea under 5 years of age from 2013 to 2018 in Beijing [34]. This difference might be due to different monitoring groups: outbreak surveillance usually focused on closed settings where people over the age of three attended, but specimens collected from sporadic cases were mainly from children under three years old who sought medical service [34]. For the lack of monitoring in other age groups, especially the elderly population, whether this difference in genotype distribution was due to the biased distribution of the monitoring population needs to be further verified. An increased proportion of GII.4 Sydney [P31] outbreaks (26.6%) was observed in 2019–2020 in China [35], and we also found that the proportion of GII.3[P12] and GII.4[P16] genotypes exceeded that of GII.2[P16] in norovirus outbreaks in 2021–2022 in Beijing, which reminded us of possible change in the prevalent strain. This study found that norovirus outbreaks of different genotypes exhibited distinct epidemiological characteristics. For instance, compared with outbreaks linked to the other three major genotypes, mixed-genotype outbreaks occurred more frequently in non-school closed settings and were primarily transmitted via the foodborne route. Outbreaks caused by GII norovirus were predominant in kindergartens, whereas the majority of GI ones took place in primary schools. Some studies found that the patients with GII.2 infections were younger and GII.2[P16] outbreaks were most frequently identified in settings where young children gathered [9,10,32], while GII.4 Sydney 2012 outbreaks were significantly associated with nursing homes/universities [10,36], and foodborne transmission was more common in GII.17[P17] outbreaks [9,32,36]. Epidemiological characteristics of norovirus outbreaks associated with different genogroups or genotypes varied across existing studies, and this is mainly attributed to the differences in the data available to each study. Furthermore, research in this field remains extremely limited to date, and the present study provides additional evidence for a more comprehensive understanding of norovirus outbreaks linked to distinct genogroups or genotypes.

Norovirus outbreaks still mainly occurred in kindergartens and primary schools [2,9,37], which was quite different from other countries, where LTCFs, including healthcare facilities and nursing homes, were the major outbreak settings [11,26,38]. The scale of norovirus outbreaks that occurred in kindergartens was relatively small, while the attack rate was high. And as the educational stage rises, the scale of norovirus outbreaks increases, while the attack rate decreases. This could be explained by the high susceptibility to norovirus infection and the contact frequency of children in the young age group. Our previous research also found that norovirus had higher transmissibility in kindergartens [39]. However, since there may be only a dozen students in a kindergarten class, the scale of norovirus outbreaks could not be very large, even if the attack rate reached one hundred percent. Studies have shown that norovirus outbreaks exhibited seasonality in cold months [10,28,37,40]. But it was not exclusively a winter preference in our study; there were small waves during March and July almost every year in Beijing. This was probably because schools were the main settings where norovirus outbreaks occurred, and the time period of March to July was the Chinese spring semester; thus outbreaks easily occurred once the source of infection was introduced and students gathered. Even though person-to-person was still the predominant transmission mode, which was consistent with other studies [10,11,37], the proportion of foodborne and waterborne outbreaks was increasing. And these outbreaks tended to be large-scale, since food and water supply usually covered a wider population. Food and water contaminated with norovirus are good carriers for transmitting norovirus, and norovirus food poisoning outbreaks are now a major public health problem worldwide [41,42,43], which also reminds us to be vigilant against foodborne and waterborne outbreaks.

In 2019–2020, most norovirus outbreaks were reported in 2019, and only a few were reported in the second half of the year, when COVID-19 erupted, and extremely strict measures were taken to curb the spread of SARS-CoV-2 (such as travel restrictions) [44], indicating that those non-specific interventions were also effective in the prevention and control of norovirus outbreaks. In 2020–2021, more targeted and less extensive measures were adopted to reduce the impact on the daily life of the public, but then the number of norovirus outbreaks showed a rebound increase. Compared with norovirus outbreaks reported when there was no COVID-19, the attack rate of norovirus outbreaks reported when a strict policy against COVID-19 was implemented (July 2020 to August 2022) was lower, which was related to the strengthened control of infectious diseases and more sensitive reporting of outbreaks during this special period of the COVID-19 pandemic. Norovirus outbreaks that were handled later tended to develop into large-scale outbreaks, providing evidence for the prompt response to curb the spread of norovirus.

There were several limitations in our study. First, our analysis relied on passive surveillance data from reported outbreaks, which may be subject to reporting bias. Mild norovirus infections and small-scale household outbreaks were inevitably underrepresented in our dataset. This underreporting may have led to an underestimation of the actual epidemic intensity of some norovirus genogroups/genotypes and the true diversity of circulating strains. Second, there is a limitation in the genotypic scope of our analysis, as we focused primarily on four major genotypes. Other less frequent or emerging genotypes were not analyzed in depth due to their low detection rates. In addition, the inference of potential shifts in prevalent norovirus strains did not account for the lack of genotype data in approximately 34% of outbreaks, which may distort the annual genotype prevalence proportions and trend characterization. It should be clarified that we comprehensively considered sample representativeness when selecting outbreaks for genotyping in practical work, which, to a certain extent, mitigated the impact of missing genotype data on the analysis of prevalent genotypes. Finally, the scope of our risk factor modeling for outbreak scale and attack rates was limited by the available variables. Our analysis was restricted to variables reported through institutional reporting mechanisms, which primarily capture facility-based factors. Consequently, our models did not account for significant external drivers such as meteorological thresholds or socio-economic disparities that may exacerbate transmission. Addressing these gaps in future research would provide a more comprehensive picture of the characteristics and trends of outbreaks driven by norovirus transmission.

## 5. Conclusions

In conclusion, norovirus outbreaks have become a significant public health issue in Beijing. Under the combined effect of herd immunity and virus mutation, the dominant genotypes of norovirus are constantly changing. The characteristics of outbreaks vary in different places; outbreaks occurred in kindergartens with high attack rates, while foodborne and waterborne transmissions or delayed outbreak response can cause large-scale outbreaks. During the COVID-19 pandemic, both the scale and attack rate of norovirus outbreaks were relatively low, which suggests that the implementation of appropriate, strict public health measures can effectively mitigate the impact of norovirus outbreaks. Based on these findings, targeted public health strategies are urgently needed, including strengthening surveillance and targeted prevention and control in kindergartens, reinforcing food safety and water hygiene management, and implementing standardized rapid response within 1 day to curb large-scale outbreaks, and sustaining molecular surveillance to monitor dynamic changes in dominant norovirus genotypes. These evidence-based measures will support local public health authorities in precise resource allocation, early warning and intervention, and will effectively reduce the disease burden of norovirus outbreaks in high-risk populations and closed settings.

## Figures and Tables

**Table 1:** Norovirus outbreaks reported in 2016–2022 in Beijing, China.

Characteristics	n (%)	Case Number		Attack Rate (%)	
		Median (IQR **)	Range	Median (IQR)	Range
Year					
Sep 2016–Aug 2017	552 (29.7)	10 (7–16)	3–217	24.2 (14.3–35.8)	0.9–80.0
Sep 2017–Aug 2018	160 (8.6)	9 (7–14)	3–127	23.8 (15.0–32.0)	2.5–100.0
Sep 2018–Aug 2019	354 (19.0)	10 (7–15)	3–259	20.0 (11.6–29.7)	0.3–75.0
Sep 2019–Aug 2020	141 (7.6)	9 (6–13)	3–389	23.8 (16.7–32.3)	0.5–72.5
Sep 2020–Aug 2021	402 (21.6)	7 (4–11)	3–92	18.0 (10.8–26.6)	0.5–100.0
Sep 2021–Aug 2022	250 (13.5)	8 (5–13)	3–324	20.8 (13.5–30.3)	1.0–65.4
Season					
Spring	847 (45.6)	9 (6–15)	3–124	21.1 (12.1–31.0)	0.5–80.0
Summer	181 (9.7)	9 (6–15)	3–324	21.2 (12.9–31.1)	0.3–75.0
Autumn	460 (24.7)	9 (6–14)	3–217	21.1 (12.9–32.8)	0.6–100.0
Winter	371 (20.0)	8 (6–14)	3–389	22.3 (13.2–32.0)	0.7–100.0
Occurrence area					
Urban district	1080 (58.1)	9 (6–14)	3–389	21.6 (13.0–32.4)	0.3–100.0
Semi-urban district	679 (36.5)	9 (6–15)	3–324	20.7 (12.5–30.0)	0.7–100.0
Rural district	100 (5.4)	9 (6–17)	3–74	19.4 (9.7–34.0)	3.0–100.0
Outbreak Setting					
Kindergarten	958 (51.5)	8 (6–12)	3–80	25.8 (17.9–36.4)	1.3–79.2
Primary school	664 (35.7)	10 (6–15)	3–217	17.5 (10.5–26.3)	0.9–72.5
Secondary school	124 (6.7)	11 (6–23)	3–140	10.0 (6.0–17.8)	0.5–66.7
College	32 (1.7)	14 (6–31)	3–389	8.8 (3.8–21.2)	0.6–71.4
Other closed settings	81 (4.4)	14 (7–23)	3–324	13.9 (6.3–28.3)	0.3–100.0
Transmission mode					
Person-to-person	1751 (94.2)	9 (6–14)	3–156	21.7 (13.3–32.0)	0.5–100.0
Foodborne	54 (2.9)	22 (14–52)	4–389	10.4 (4.4–20.0)	0.7–100.0
Waterborne	6 (0.3)	31 (19–205)	13–324	1.3 (0.7–1.4)	0.3–6.1
Unknown	48 (2.6)	6 (4–9)	3–46	14.6 (8.3–27.3)	3.8–78.6
Time period of COVID-19 *					
Before COVID-19	1199 (64.5)	10 (7–15)	3–389	22.5 (13.5–33.3)	0.3–100.0
Extremely strict policy against COVID-19	8 (0.4)	11 (9–15)	6–31	25.3 (17.7–32.5)	14.7–35.0
Early phase of the strict policy against COVID-19	224 (12.1)	7 (4–10)	3–57	17.2 (11.8–26.6)	3.0–100.0
The latter phase of the strict policy against COVID-19	428 (23.0)	8 (5–12)	3–324	20.0 (11.6–29.3)	0.5–65.4

* Before COVID-19 is the time period from 1 September 2016 to 31 December 2019; extremely strict policy against COVID-19 is the time period from 1 January 2020 to 30 June 2020; strict policy against COVID-19 is the time period from 1 July 2020 to 31 August 2022. ** IQR is the abbreviation of interquartile range.

**Table 2:** Epidemiological characteristics of norovirus outbreaks by major genogroups and main genotypes reported in Beijing, 2016–2022.

Characteristics	Genogroup n (%)				Genotype n (%)				
	GII	GI	GI/GII Mixed	*p*	GII.2[P16]	GII.3[P12]	GII.4[P31]	Mixed-Genotype	*p*
Year				<0.001					<0.001
Sep 2016–Aug 2017	538 (33.9)	6 (3.3)	3 (5.4)		350 (55.1)	9 (8.6)	8 (10.4)	12 (19.4)	
Sep 2017–Aug 2018	124 (7.8)	25 (14.0)	6 (10.7)		36 (5.7)	6 (5.7)	11 (14.3)	1 (1.6)	
Sep 2018–Aug 2019	258 (16.2)	58 (32.4)	30 (53.6)		98 (15.4)	22 (20.9)	15 (19.5)	13 (20.9)	
Sep 2019–Aug 2020	124 (7.8)	9 (5.0)	6 (10.7)		31 (4.9)	3 (2.9)	13 (16.9)	1 (1.6)	
Sep 2020–Aug 2021	361 (22.7)	30 (16.8)	4 (7.1)		117 (18.4)	18 (17.1)	14 (18.2)	28 (45.2)	
Sep 2021–Aug 2022	184 (11.6)	51 (28.5)	7 (12.5)		3 (0.5)	47 (44.8)	16 (20.7)	7 (11.3)	
Season				<0.001					<0.001
Spring	714 (44.9)	102 (57.0)	19 (33.9)		314 (49.4)	33 (31.4)	21 (27.3)	25 (40.4)	
Summer	140 (8.8)	22 (12.3)	17 (30.4)		66 (10.4)	5 (4.8)	4 (5.2)	9 (14.5)	
Autumn	392 (24.7)	43 (24.0)	13 (23.2)		170 (26.8)	24 (22.8)	35 (45.4)	10 (16.1)	
Winter	343 (21.6)	12 (6.7)	7 (12.5)		85 (13.4)	43 (41.0)	17 (22.1)	18 (29.0)	
Occurrence area				0.49					0.36
Urban district	937 (59.0)	103 (57.5)	29 (51.8)		354 (55.7)	54 (51.4)	47 (61.0)	35 (56.5)	
Semi-urban district	563 (35.4)	70 (39.1)	23 (41.1)		254 (40.0)	49 (46.7)	30 (39.0)	24 (38.7)	
Rural district	89 (5.6)	6 (3.4)	4 (7.1)		27 (4.3)	2 (1.9)	0 (0.0)	3 (4.8)	
Outbreak Setting				<0.001					<0.001
Kindergarten	878 (55.3)	43 (24.0)	14 (25.0)		329 (51.8)	87 (82.9)	59 (76.6)	33 (53.2)	
Primary school	536 (33.7)	102 (57.0)	16 (28.6)		247 (38.9)	18 (17.1)	14 (18.2)	15 (24.2)	
Secondary school	100 (6.3)	16 (8.9)	7 (12.5)		41 (6.5)	0 (0.0)	2 (2.6)	4 (6.5)	
College	24 (1.5)	6 (3.4)	1 (1.8)		7 (1.1)	0 (0.0)	1 (1.3)	1 (1.6)	
Other closed settings	51 (3.2)	12 (6.7)	18 (32.1)		11 (1.7)	0 (0.0)	1 (1.3)	9 (14.5)	
Transmission mode				<0.001					0.01
Person-to-person	1516 (97.6)	161 (93.1)	45 (83.3)		619 (99.0)	102 (98.1)	74 (97.4)	57 (93.4)	
Foodborne	33 (2.1)	12 (6.9)	7 (13.0)		6 (1.0)	2 (1.9)	2 (2.6)	4 (6.6)	
Waterborne	4 (0.3)	0 (0.0)	2 (3.7)		0 (0.0)	0 (0.0)	0 (0.0)	0 (0.0)	

**Table 3:** Risk factors associated with the scale of norovirus outbreaks were established through the establishment of a multivariable logistic regression model.

Factor	Scale of Norovirus Outbreaks		Univariable Analysis		Multivariable Analysis	
	≥15 Cases	<15 Cases	Crude OR (95% CI)	*p*	Adjusted OR (95% CI)	*p*
Year						
Sep 2016–Aug 2017	175 (31.7)	377 (68.3)	Ref		Ref	
Sep 2017–Aug 2018	39 (24.4)	121 (75.6)	0.694 (0.460–1.031)	0.076	0.704 (0.450–1.085)	0.118
Sep 2018–Aug 2019	106 (29.9)	248 (70.1)	0.921 (0.688–1.229)	0.576	0.729 (0.521–1.015)	0.063
Sep 2019–Aug 2020	29 (20.6)	112 (79.4)	0.558 (0.352–0.861)	0.010	0.552 (0.335–0.886)	0.016
Sep 2020–Aug 2021	64 (15.9)	338 (84.1)	0.408 (0.294–0.560)	<0.001	0.370 (0.258–0.525)	<0.001
Sep 2021–Aug 2022	54 (21.6)	196 (78.4)	0.594 (0.415–0.838)	0.004	0.563 (0.366–0.856)	0.008
Season						
Spring	220 (26.0)	627 (74.0)	Ref			
Summer	46 (25.4)	135 (74.6)	0.971 (0.667–1.394)	0.876		
Autumn	112 (24.3)	348 (75.7)	0.917 (0.704–1.191)	0.519		
Winter	89 (24.0)	282 (76.0)	0.899 (0.675–1.191)	0.464		
Occurrence area						
Urban district	262 (24.3)	818 (75.7)	Ref		Ref	
Semi-urban district	171 (25.2)	508 (74.8)	1.051 (0.840–1.312)	0.661	1.017 (0.798–1.293)	0.894
Rural district	34 (34.0)	66 (66.0)	1.608 (1.030–2.472)	0.033	1.595 (0.982–2.552)	0.055
Outbreak setting						
Kindergarten	168 (17.5)	790 (82.5)	Ref		Ref	
Primary school	198 (29.8)	466 (70.2)	1.998 (1.580–2.530)	<0.001	1.922 (1.495–2.474)	<0.001
Secondary school	50 (40.3)	74 (59.7)	3.177 (2.131–4.709)	<0.001	3.245 (2.104–4.982)	<0.001
College	15 (46.9)	17 (53.1)	4.149 (2.009–8.489)	<0.001	4.016 (1.792–8.822)	<0.001
Other closed settings	36 (44.4)	45 (55.6)	3.762 (2.344–6.004)	<0.001	2.077 (1.091–3.834)	0.022
Transmission mode						
Person-to-person	418 (23.9)	1333 (76.1)	Ref		Ref	
Foodborne	40 (74.1)	14 (25.9)	9.111 (5.029–17.497)	<0.001	7.987 (4.076–16.474)	<0.001
Waterborne	5 (83.3)	1 (16.7)	15.944 (2.562–305.948)	0.012	19.109 (2.660–390.181)	0.011
Unknown	4 (8.3)	44 (91.7)	0.290 (0.087–0.720)	0.018	0.240 (0.070–0.619)	0.008
Genogroup						
GII	382 (24.0)	1207 (76.0)	Ref			
GI	53 (29.6)	126 (70.4)	1.329 (0.939–1.859)	0.102		
GI/GII mixed	19 (33.9)	37 (66.1)	1.623 (0.905–2.820)	0.093		
Others	13 (37.1)	22 (62.9)	1.867 (0.907–3.693)	0.078		
Genotype						
GII.2[P16]	182 (28.7)	453 (71.3)	Ref			
GII.3[P12]	28 (26.7)	77 (73.3)	0.905 (0.560–1.426)	0.6746		
GII.4[P31]	12 (15.6)	65 (84.4)	0.460 (0.232–0.841)	0.0171		
Mixed-genotype	17 (27.4)	45 (72.6)	0.940 (0.511–1.656)	0.8363		
others	228 (23.3)	752 (76.7)	0.755 (0.602–0.947)	0.015		
Time period of COVID-19 *						
Before COVID-19	347 (28.9)	852 (71.1)	Ref			
Extremely strict policy against COVID-19	2 (25.0)	6 (75.0)	0.818 (0.120–3.572)	0.807		
Strict policy against COVID-19	118 (18.1)	534 (81.9)	0.543 (0.428–0.685)	<0.001 **		
Response timeliness						
Early (≤1 day)	176 (21.7)	634 (78.3)	Ref		Ref	
Medium (2–3 days)	220 (28.1)	564 (71.9)	1.405 (1.119–1.767)	0.00351	1.481 (1.160–1.893)	0.002
Late (>3 days)	71 (26.8)	194 (73.2)	1.318 (0.955–1.808)	0.08953	1.186 (0.833–1.675)	0.338

* Before COVID-19 is the time period from 1 September 2016 to 31 December 2019; the extremely strict policy against COVID-19 is the time period from 1 January 2020 to 30 June 2020; the strict policy against COVID-19 is the time period from 1 July 2020 to 31 August 2022. ** The category “Strict policy against COVID-19” within the variable time period of COVID-19 exhibited multicollinearity with the categories “2020–2021” and “2021–2022” in the variable surveillance year. Therefore, the variable time period of COVID-19 was not included in the multivariable analysis.

**Table 4:** Risk factors associated with the attack rate of norovirus outbreaks were established through the establishment of a multivariable logistic regression model.

Factor	Attack Rate of Norovirus Outbreaks		Univariate Analysis		Multivariate Analysis	
	≥30.0%	<30.0%	Crude OR (95% CI)	*p*	Adjusted OR (95% CI)	*p*
Year						
Sep 2016–Aug 2017	193 (35.0)	359 (65.0)	Ref		Ref	
Sep 2017–Aug 2018	46 (28.8)	114 (71.2)	0.751 (0.507–1.096)	0.144	0.801 (0.523–1.214)	0.301
Sep 2018–Aug 2019	88 (24.9)	266 (75.1)	0.615 (0.456–0.827)	0.001	0.766 (0.546–1.069)	0.119
Sep 2019–Aug 2020	47 (33.8)	92 (66.2)	0.950 (0.638–1.401)	0.799	1.101 (0.710–1.645)	0.663
Sep 2020–Aug 2021	85 (21.4)	313 (78.6)	0.505 (0.374–0.678)	<0.001	0.486 (0.351–0.671)	<0.001
Sep 2021–Aug 2022	66 (26.7)	181 (73.3)	0.678 (0.485–0.942)	0.022	0.667 (0.446–0.991)	0.047
Season						
Spring	231 (27.4)	613 (72.6)	Ref			
Summer	48 (26.7)	132 (73.3)	0.965 (0.666–1.379)	0.847		
Autumn	138 (30.0)	322 (70.0)	1.137 (0.884–1.459)	0.314		
Winter	108 (29.5)	258 (70.5)	1.111 (0.846–1.454)	0.447		
Occurrence area						
Urban district	324 (30.1)	753 (69.9)	Ref		Ref	
Semi-urban district	173 (25.7)	501 (74.3)	0.803 (0.646–0.995)	0.046	0.708 (0.559–0.895)	0.004
Rural district	28 (28.3)	71 (71.7)	0.917 (0.572–1.431)	0.708	1.172 (0.709–1.899)	0.526
Outbreak setting						
Kindergarten	376 (39.2)	582 (60.8)	Ref		Ref	
Primary school	120 (18.1)	543 (81.9)	0.342 (0.269–0.432)	<0.001	0.325 (0.252–0.416)	<0.001
Secondary school	6 (4.8)	118 (95.2)	0.079 (0.031–0.166)	<0.001	0.081 (0.031–0.173)	<0.001
College	4 (13.8)	25 (86.2)	0.248 (0.073–0.644)	0.010	0.309 (0.089–0.830)	0.035
Other closed settings	19 (25.0)	57 (75.0)	0.516 (0.295–0.865)	0.015	0.998 (0.523–1.860)	0.994
Transmission mode						
Person-to-person	508 (29.1)	1238 (70.9)	Ref			
Foodborne	7 (13.2)	46 (86.8)	0.371 (0.152–0.775)	0.015		
Waterborne	0 (0.0)	5 (100.0)	0.000 (NA)	0.972		
Unknown	10 (21.7)	36 (78.3)	0.677 (0.316–1.323)	0.280		
Genogroup						
GII	481 (30.4)	1101 (69.6)	Ref		Ref	
GI	26 (14.6)	152 (85.4)	0.392 (0.250–0.591)	<0.001	0.710 (0.435–1.124)	0.156
GI/GII mixed	6 (10.9)	49 (89.1)	0.280 (0.107–0.609)	0.004	0.349 (0.128–0.799)	0.022
Others	12 (34.3)	23 (65.7)	1.194 (0.571–2.376)	0.622	1.340 (0.614–2.808)	0.446
Genotype						
GII.2[P16]	213 (33.6)	421 (66.4)	Ref		Ref	
GII.3[P12]	45 (42.9)	60 (57.1)	1.482 (0.970–2.252)	0.066	1.331 (0.826–2.138)	0.238
GII.4[P31]	20 (26.0)	57 (74.0)	0.694 (0.397–1.166)	0.180	0.551 (0.304–0.965)	0.042
Mixed-genotype	16 (26.2)	45 (73.8)	0.703 (0.377–1.247)	0.244	0.804 (0.416–1.495)	0.502
Others	231 (23.7)	742 (76.3)	0.615 (0.493–0.768)	<0.001	0.733 (0.563–0.955)	0.021
Time period of COVID-19 *						
Before COVID-19	371 (30.9)	828 (69.1)	Ref			
Extremely strict policy against COVID-19	3 (50.0)	3 (50.0)	2.232 (0.411–12.108)	0.327		
Strict policy against COVID-19	151 (23.4)	494 (76.6)	0.682 (0.547–0.848)	<0.001 **		
Response timeliness						
Early (≤1 day)	213 (26.4)	593 (73.6)	Ref			
Medium (2–3 days)	230 (29.5)	549 (70.5)	1.166 (0.937–1.453)	0.170		
Late (>3 days)	82 (30.9)	183 (69.1)	1.247 (0.918–1.687)	0.154		

* Before COVID-19 is the time period from 1 September 2016 to 31 December 2019; the extremely strict policy against COVID-19 is the time period from 1 January 2020 to 30 June 2020; the strict policy against COVID-19 is the time period from 1 July 2020 to 31 August 2022. ** The category “Strict policy against COVID-19” within the variable time period of COVID-19 exhibited multicollinearity with the categories “2020–2021” and “2021–2022” in the variable surveillance year. Therefore, the variable time period of COVID-19 was not included in the multivariable analysis.

## Data Availability

Data not publicly available.
